# Effects of oral carbohydrate with amino acid solution on the metabolic status of patients in the preoperative period: a randomized, prospective clinical trial

**DOI:** 10.1007/s00540-016-2217-y

**Published:** 2016-07-20

**Authors:** Rie Tsutsumi, Nami Kakuta, Takako Kadota, Takuro Oyama, Katsuyoshi Kume, Eisuke Hamaguchi, Noriko Niki, Katsuya Tanaka, Yasuo M. Tsutsumi

**Affiliations:** 1 Department of Nutrition, Tokushima University, Tokushima, Japan; 2 Department of Anesthesiology, Tokushima University, Tokushima, Japan

**Keywords:** Enhanced recovery after surgery, Preoperative carbohydrate, Quality of life, Surgical outcomes, Nutritional status

## Abstract

**Objective:**

Enhanced recovery after surgery is increasingly desired nowadays, and preoperative nutrient intake may be beneficial for this purpose. In this study, we investigated whether the intake of preoperative carbohydrate with amino acid (ONS) solution can improve starvation status and lipid catabolism before the induction of anesthesia.

**Methods:**

This randomized, prospective clinical trial included 24 patients who were divided into two groups before surgery under general anesthesia: a control group, comprising patients who fasted after their last meal the day before surgery (permitted to drink only water), and an ONS group, comprising patients who consumed ONS solution 2 h before surgery. Biochemical markers, the respiratory quotient, and psychosomatic scores were assessed at the initiation of anesthesia.

**Results:**

Compared with the control group, the ONS group showed significantly lower serum free fatty acid levels [control group: 828 (729, 1004) µEq/L, ONS group: 479 (408, 610) µEq/L, *P* = 0.0002, median (25th, 75th percentile)] and total ketone bodies [control group: 119 (68, 440) µmol/L, ONS group: 40 [27, 64] µmol/L, *P* = 0.037]. In addition, analysis using the Visual Analog Scale showed higher preoperative scores for anxiety, hunger, and thirst for the control group, with no differences in any other measure of subjective well-being between groups.

**Conclusions:**

The results of this study suggest that preoperative ONS intake improves lipid catabolism and starvation status before the induction of anesthesia. Furthermore, it can provide better preoperative mental health compared with complete fasting.

## Introduction

Enhanced recovery after surgery (ERAS) represents a multimodal approach to improving the outcomes of medical treatment and care [1]. Preoperative intake of oral carbohydrates for ERAS has resulted in some benefits [2, 3]. In patients undergoing surgery, the intake of oral carbohydrates the night before surgery or 2 h before anesthesia was found to decrease postoperative insulin resistance, with a slight decrease in insulin-stimulated glucose disposal [4]. Carbohydrate intake also decreases preoperative discomfort, such as thirst, hunger, and anxiety [5, 6], and postoperative discomfort, as demonstrated in colorectal patients, in addition to decreasing the length of postoperative hospital stay and accelerating the return of gastrointestinal function and grip strength [2]. However, some studies showed that preoperative carbohydrate intake did not decrease the length of hospital stay or improve insulin resistance [7–9]. Therefore, it remains unclear whether preoperative carbohydrate intake is beneficial to patients.

There are some clinical reports of the benefit of carbohydrate loading before surgery using 12.6 % carbohydrates [10, 11]. However, we typically use 18 % carbohydrate drink with amino acid, instead of beverages containing 12.6 % carbohydrates which are not available in Japan [12–15]. Although the effects of drinks containing 18 % carbohydrates and amino acid [oral nutritional supplement (ONS)] on insulin sensitivity and glucose metabolism have been evaluated [15], their influence on lipid or protein catabolism and energy intake/expenditure before surgery remains unclear. In addition, studies on ERAS interventions followed a multimodal enhanced protocol, and it is unclear whether this protocol included any carbohydrate/protein-based intervention [1]. Therefore, the primary objective of this study was to determine whether the preoperative intake of ONS solution can improve the starvation status and lipid catabolism before the induction of anesthesia.

## Materials and methods

### Patients and study protocol

This study was approved by the Human Research Ethics Committee of the Tokushima University and Sikoku Central Hospital and was registered in a clinical trials database (UMIN000013778). Written informed consent was obtained from all patients, and the study was conducted in accordance with the principles outlined in the Declaration of Helsinki.

Inclusion criteria were established: ages were between 20 and 90 years, the patient was undergoing minor surgery under general anesthesia, and had an American Society of Anesthesiologists (ASA) physical status (PS) of I to III. Obese [body mass index (BMI) >30 kg/m^2^] and emaciated (BMI <17 kg/m^2^) patients and patients with diabetes were excluded.

**Fig. 1:**
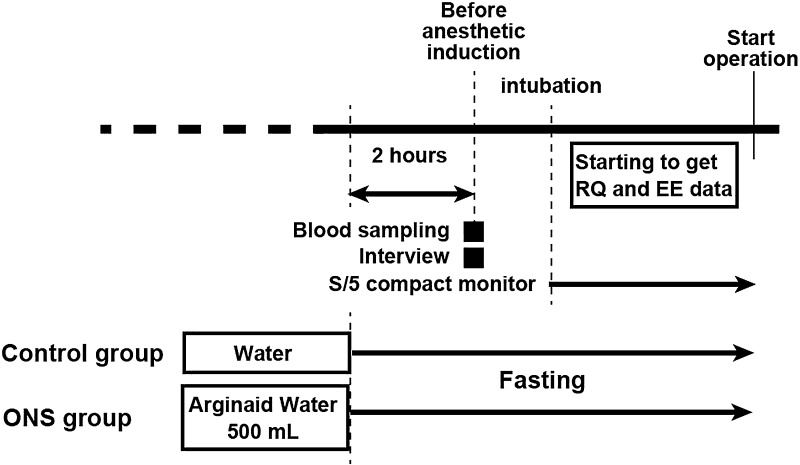
Study protocol. *RQ* respiratory quotient, *EE* energy expenditure, *ONS* oral nutritional supplement

To ensure blinding of the investigators, a statistician not involved in the clinical study generated the randomization schedule using computer-generated distribution (QuickCalcs, GraphPad Inc., La Jolla, CA, USA). On the day before surgery, the 24 patients were randomized to one of two groups (Fig. 1) and the primary anesthesiologist supplied ONS to the patients to blind the anesthesia team: a control group, comprising 12 patients who fasted after 21:00 on the day before surgery (permitted to drink only water), and an ONS group, comprising 12 patients who received 500 mL of ONS (Arginaid Water^®^; Nestle Japan, Kobe, Japan) 2 h before surgery. The nutrient profiles of the carbohydrate drinks are shown in Table 1.

**Table 1:** Major nutrients in carbohydrate formula composition

Nutrients	Arginaid water 100 mL
Calories (kcal)	80
Arginine (g)	2.0
CHO (g)	18
Fat (g)	0
Sodium (mg)	0
Phosphate (mg)	140
Zinc (mg)	
Manganese (mg)	0
Copper (mg)	0.8
Osmotic pressure (mOsm/L)	560–580

No patient was premedicated. Upon arrival in the operating room, a 20G catheter was inserted into the left or right forearm of each patient and bicarbonate Ringer’s solution was infused for 1 h. General anesthesia was induced and the patients were intubated. The tidal volume was set at 7 mL/kg, the respiratory rate at 10/min, and the O_2_/air mixture at an FiO_2_ of 0.5. Sampling tubes were connected with a Datex-Ohmeda S/5 compact monitor (GE Healthcare, Helsinki, Finland). It took approximately 20 min for the data on the S/5 compact monitor to stabilize.

### Measurements

Blood samples were obtained before the induction of anesthesia and centrifuged at 150*g* at 4 °C for 10 min (Table Top cooling centrifuge 2800, Kubota, Tokyo, Japan). The plasma and serum samples were stored at −20 °C until analysis and were analyzed by a clinical laboratory testing company (SRL Inc., Tokyo, Japan). The respiratory quotient (RQ) and energy expenditure (EE) were measured by the S/5 compact monitor.

Participants completed a 100-mm Visual Analog Scale (VAS; 0, “not at all”; 100, “extreme”) to measure subjective well-being upon entering the operating room. The variables examined were those measured in a previous study [6]. In addition, the Patient Health Questionnaire-9 (PHQ-9), a multiple instrument for screening, diagnosing, monitoring, and measuring the severity of mental health, was administered. This tool rates the frequency of symptoms, which is then factored into the scoring severity index. The PHQ-9 is completed by the patients and scored by the clinician. The questions pertain to nine items: little interest or pleasure in doing things, feeling low, trouble falling asleep, feeling tired, poor appetite, feeling bad about oneself, trouble concentrating on things, moving or speaking slowly, and suicidal or self-harming thoughts [16, 17].

### Statistical analysis

The size of the study was chosen to detect a difference in the concentration of ketone bodies. A previous study reported that the concentration of ketone bodies in volunteers who received preoperative oral carbohydrates decreased from 124 ± 118 to 22 ± 4 mmol/L before the induction of anesthesia [15]. Therefore, the required number of patients in each group was 12, with an alpha of 0.05 and a power of 80 % for ketone bodies (SPSS SamplePower, IBM Co., Armonk, NY, USA). Comparisons between groups were performed using Student’s *t* test or the Mann–Whitney *U* test. A *P* value of <0.05 was considered statistically significant.

## Results

**Fig. 2:**
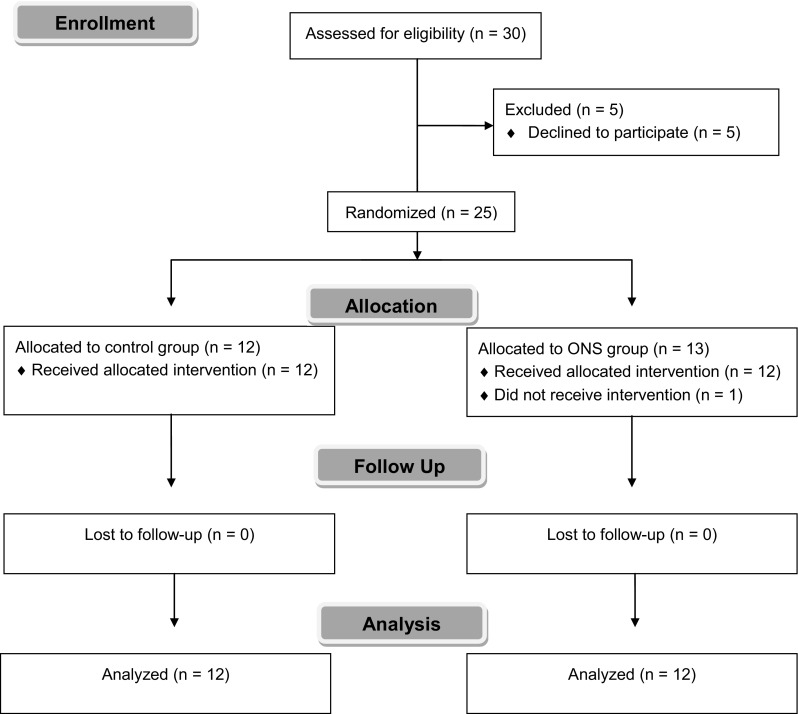
CONSORT flow chart showing the selection process for patients

**Fig. 3:**
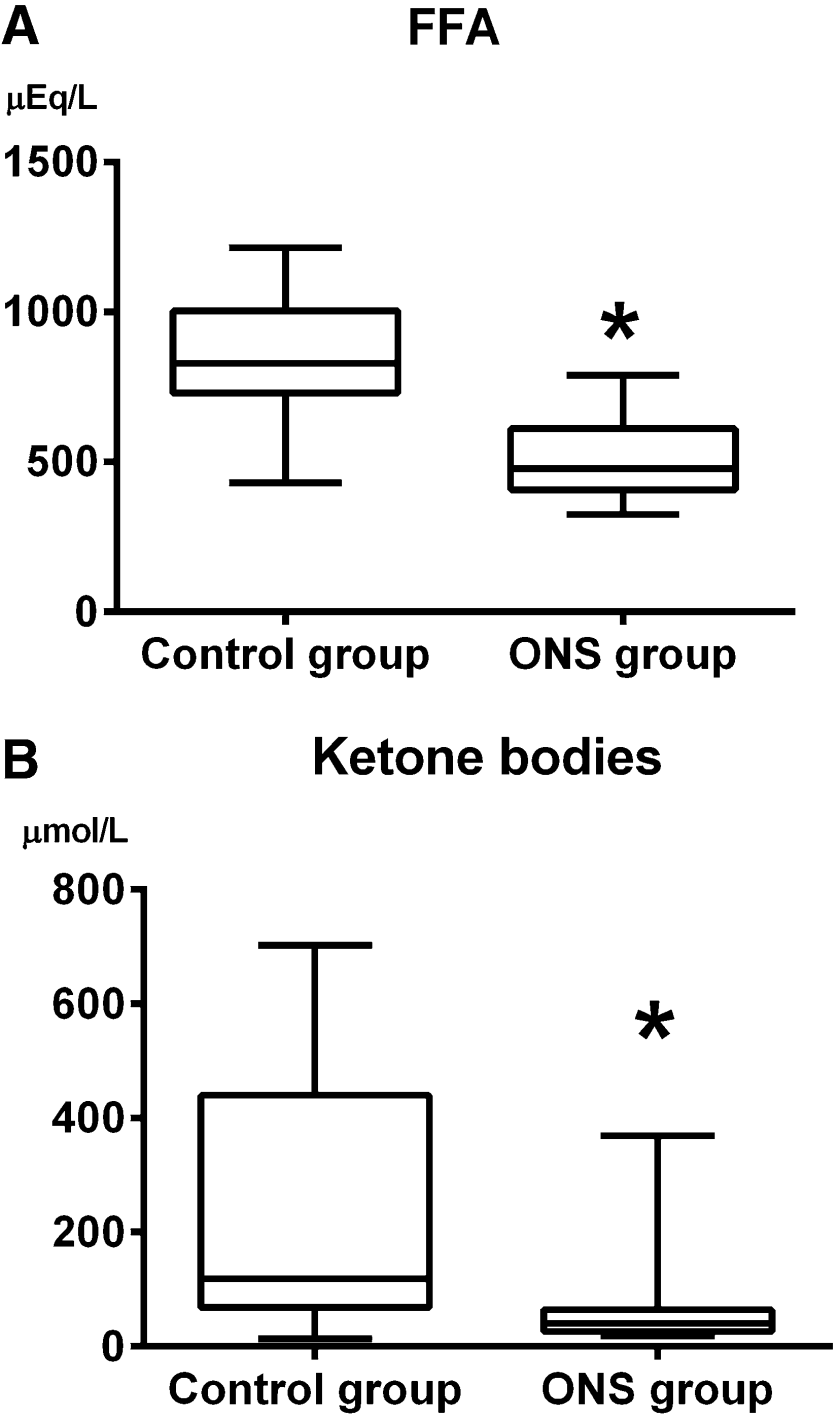
Serum concentration of free fatty acids (**a**) and total ketone bodies (**b**) at the initiation of anesthesia. The *boxes* represent the 25th to 75th percentiles, and the *horizontal lines* within the *boxes* represent median values. The *whiskers* represent the lowest and highest values in the 25th percentile minus 1.5 interquartile range (IQR) and 75th percentile plus 1.5 IQR regions, respectively. *P < 0.05 compared with the control group

The mean age of the 24 patients (16 men, 8 women) was 43 (21–63) years. One ONS group patient was excluded because of failure to consume the carbohydrate drink (Fig. 2). The demographic characteristics (Table 2) and preoperative total fluid intake [control group: 450 (250, 750) mL, ONS group: 600 (500, 738) mL, *P* = 0.085, median (25th, 75th percentile)] did not differ significantly between groups. Compared with the control group, the ONS group showed significantly lower serum free fatty acid (FFA) levels [control group: 828 (729, 1004) µEq/L, ONS group: 479 (408, 610) µEq/L, *P* = 0.0002, median (25th, 75th percentile); Fig. 3a] and total ketone bodies [control group: 119 (68, 440) µmol/L, ONS group: 40 [27, 64] µmol/L, *P* = 0.037, median (25th, 75th percentile); Fig. 3b].

**Table 2:** Characteristics of patients at onset of surgery

	Control group	ONS group	*P* value
Age (years)	43 (21–61)	43 (29–63)	0.723
Male:female (*n*)	8:4	8:4	
Baseline body mass index (kg/m^2^)	23.9 ± 3.0	23.2 ± 3.3	0.620
ASA Performance Status (*n*)			
I	4	5	0.705
II	8	7	
Type of surgery (*n*)			
Salpingo-oophorectomy	1	2	
Partial mastectomy	2	1	
Endoscopic sinus surgery	2	2	
Skin surgery	4	5	
Arthroscopic surgery	3	2	

Ranges in *parentheses*

The preoperative serum glucose, insulin, 3-methylhistidine (3-MH), creatinine (Cr), and acute-phase protein levels and inflammatory indexes are shown in Table 3. There was no significant difference in any of these parameters between the two groups. Whole body oxygen consumption, carbon dioxide production, and EE were similar between groups after intubation, although RQ was lower in the control group than in the ONS group (Table 4).

**Table 3:** Physiological data

	Control group	ONS group	*P* value
Glucose (mg/dL)	110 ± 11	107 ± 9	0.525
Insulin (μIU/mL)	3.4 ± 2.4	4.9 ± 3.0	0.199
3-MH (nmol/mL)	3.3 ± 0.6	3.1 ± 1.1	0.586
Creatinine (mg/dL)	0.58 ± 0.17	0.60 ± 0.16	0.757
3-MH/creatinine ratio	6.1 ± 2.1	5.2 ± 1.9	0.210
Prealbumin (g/L)	18.1 ± 6.7	19.1 ± 4.1	0.663
CRP (mg/L)	0.38 ± 0.22	0.33 ± 0.18	0.570
Albumin (g/dL)	3.1 ± 0.5	3.3 ± 0.5	0.271

*3-MH* 3-methylhistidine, *CRP* C-reactive protein

**Table 4:** Gaseous exchange and energy expenditure

	Control group	ONS group	*P* value
Oxygen consumption (mL/min)	221 ± 38	209 ± 33	0.444
Carbon dioxide production (mL/min)	171 ± 35	179 ± 31	0.587
Respiratory quotient	0.78 ± 0.08	0.85 ± 0.08	0.024
Energy expenditure (kcal/day)	1189 ± 263	1224 ± 224	0.730

**Fig. 4:**
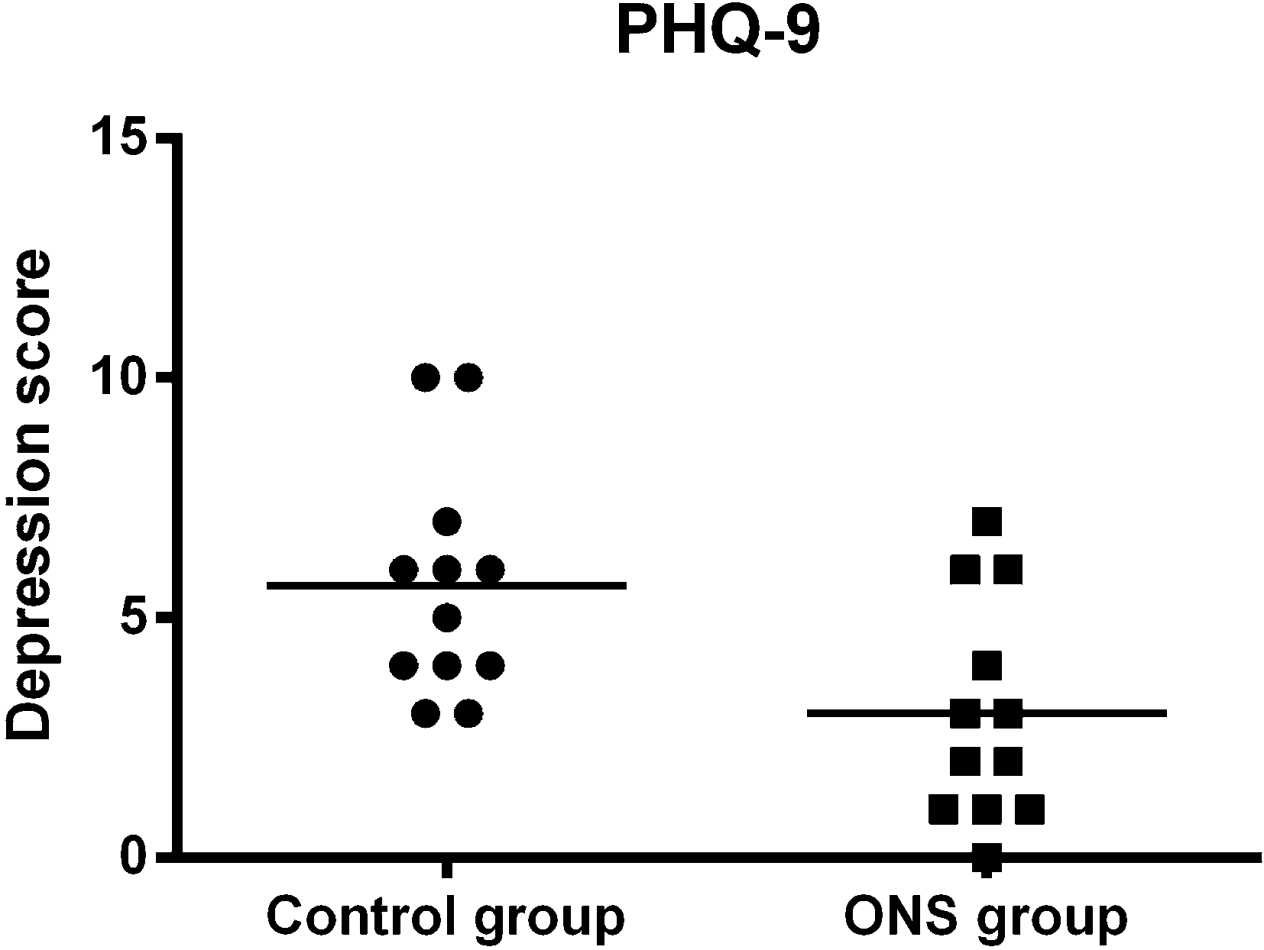
Patient Health Questionnaire-9 (PHQ-9) scores. Values are reported as medians (*center lines*). The ONS group shows significantly lower scores (*P* = 0.013)

The preoperative VAS scores for anxiety, hunger, and thirst were higher in the control group than in the ONS group, with no differences in any other measure of subjective well-being between groups (Table 5). In addition, the PHQ-9 score was significantly lower in the ONS group than in the control group, indicating a better preoperative quality of life (QOL) in the former than in the latter (Fig. 4).

**Table 5:** Preoperative visual analog scale score

	Control group	ONS group	*P* value
Anxiety	32 (22, 45)	22 (15, 24)	0.048
Depression	23 (10, 39)	20 (11, 29)	0.73
Hunger	43 (30, 59)	13 (3, 20)	0.001
Malaise	28 (20, 38)	20 (16, 30)	0.35
Nausea	0 (0, 9)	0 (0, 9)	0.84
Thirst	28 (13, 30)	12 (10, 15)	0.01

Ranges in *parentheses*

## Discussion

The results of this study suggest that preoperative ONS intake decreases the levels of FFA and ketone bodies, thus attenuating lipid catabolism before surgery, with the decreased RQ in the control group supporting this fact. Furthermore, preoperative ONS intake resulted in improved well-being compared with complete fasting.

Beverages containing 12.6 % carbohydrates, which are common preoperative supplements in Europe, are not available in Japan, so we used a readily available commercial drink (18 % carbohydrate and 2 % arginine; Arginaid Water^®^). Although some clinical studies report the benefits of 12.6 % carbohydrate loading of 800 mL the evening before the surgery and 400 mL 2 h before the anesthesia [10, 11], there are few reports showing the benefit of preoperative 18 % carbohydrates for preoperative conditions such as starvation, lipid catabolism, and patient’s QOL. Because most Japanese patients find it hard to drink a high volume, a small size (100 kcal/125 mL/package) is suited to them [15].

The patients included in the present study represent those who are typically allowed to drink clear fluids up to 2 h before surgery, according to existing guidelines [18–20]. There was no adverse event in the present study, but the rate of gastric emptying might be slower with clear fluid containing carbohydrate and amino acid [21]. These patients also exhibited an ASA PS of I–II; more than 85 % patients in our hospital belong to this category. Therefore, preoperative preparation with ONS is potentially relevant and suitable for the majority of patients undergoing elective surgery.

A number of randomized controlled trials have examined whether preoperative carbohydrate intake results in improved postoperative recovery. However, several randomized trials including patients undergoing major abdominal [7, 8, 12, 22] or cardiac surgery [23] did not show improved clinical outcomes with preoperative carbohydrate intake. The median duration of hospital stay was 8 days in the carbohydrate group and 10 days in the placebo group in one study [7], while it was 17 and 16 days, respectively, in another study involving cardiac surgery patients [23]. In contrast, Noblett et al. [2] showed that the median hospital stay after colorectal surgery was significantly lower for patients who consumed carbohydrate drinks before surgery than for those who consumed water. Therefore, in the present study, the postoperative clinical outcomes (with or without ONS) were not assessed because the study focused on the potential clinical effects of ONS in the preoperative period. In addition, our preoperative data were not affected by anesthesia protocols (general/epidural anesthesia), perioperative fluid, surgical duration, surgical models (major, minor, or laparoscopic), and stress hormones and cytokines, the effects of which are likely to carry over to the postoperative phase. However, by way of precaution, a surgical model that induced minimal surgical stress (minor surgery) was selected to evaluate the effects of preoperative fasting compared with those of feeding per se, without the confounding effects of mental anxiety related to major surgery.

Food restriction is usually enforced from midnight before the day of surgery. We hypothesized that 200 kcal (250 mL) ONS is appropriate as late evening intake. Glycogen stores deplete rapidly when patients are starved, thereby increasing the demand for amino acids for gluconeogenesis, rather than for tissue repair [24–26]. Furthermore, prolonged fasting has been demonstrated to decrease whole body protein synthesis, thus potentially accentuating protein catabolism after major surgery [27]. This is the most marked in the first few days following surgery and has been related to postoperative complications and length of hospital stay. In addition, investigators have targeted 2 h as the interval between carbohydrate ingestion and induction. Insulin levels have been reported to return to near-baseline values 2 h after oral carbohydrate intake, although insulin action is nevertheless enhanced beyond this period [28, 29]. In the present study, serum lipid catabolism (FFA and ketone bodies) was significantly lower in the ONS group than in the control group before surgical stress.

RQ is the indicator of which nutrient substrate is being metabolized to supply the body with energy. The RQ of the patients in ONS group was 0.85, implying that the administered ONS was used accurately for energy expenditure without exhaustion and glycogen storage in the liver was fully maintained during the preoperative period. The RQ of 0.78 in the control group indicated that fat was the predominant fuel source, suggesting that serum lipid catabolism might be occurring, whereas RQ levels in ONS group confirmed that lipid catabolism was inhibited (Fig. 3).

Studies have shown that formulae using acute-phase proteins may predict risks for hospitalized patients [13, 30, 31]; similar findings were observed in the two groups in the present study. C-reactive protein (CRP) is a positive acute-phase protein, the levels of which correlate well with the intensity of inflammation. On the other hand, decreased levels of negative acute-phase proteins such as albumin and prealbumin after trauma are expected because of the inhibition of their synthesis by proinflammatory cytokines.

Our results have shown that preoperative ONS intake is beneficial for not only decreasing the fasting time but also improving the preoperative QOL. Preoperative anxiety, hunger, and thirst were attenuated in the ONS group, as opposed to the findings of Henriksen et al. [32]. However, our findings were consistent with those in other previous studies [6, 33, 34]. Furthermore, the ONS group showed lower PHQ-9 scores, indicating superior well-being and fewer general psychosomatic problems.

The commercial carbohydrate drinks with amino acid (Arginaid Water^®^) used in the present study are common and frequently used as preoperative oral fluid supplements in Japan. The carbohydrate content is 18 %, much higher than in the fluid (preOp^®^, Nutricia, Danone, Paris, France) most commonly used before surgery in Europe and other countries; however, there were no episodes of hyperglycemia or nausea/vomiting caused by the high carbohydrate content. Additionally, we could not determine the contribution of amino acid. Arginine has been reported to affect disposable insulin-mediated glucose [35] and to regulate metabolism of energy substrate [36]. In our study, however, there were no significant between-group differences in energy expenditure. Therefore, we could not explain the effects of 2 % arginine on energy metabolism and lipid catabolism from our findings.

Other limitations of this study include the fact that the aforementioned beneficial effects cannot be solely attributed to one of the constituents of the drink (carbohydrate and arginine). Further studies would need to investigate the contributions of the different constituents of the drink to any potential beneficial effects observed. Furthermore, the present study did not measure the relative changes in insulin sensitivity, although the effects of preoperative fasting and carbohydrate drink intake on this parameter have been well characterized in previous studies [37, 38]. Furthermore, we did not include elderly patients, high-risk patients, or patients with an ASA PS of III or higher, and the safety and efficacy of preoperative ONS intake in these patients remain unclear. These patients had low BMI and low muscle mass, so that their glucose store should be less than in healthy subjects., More benefits of ONS loading were therefore expected. Further study is needed to evaluate the efficacy of ONS for these patients.

## Conclusions

In conclusion, the results of this study suggest that preoperative ONS intake can improve lipid catabolism and starvation status before the induction of anesthesia, also resulting in better mental health compared with that after complete fasting.
